# Cytotoxicity and Genome Characteristics of an Emetic Toxin-Producing *Bacillus cereus* Group sp. Isolated from Raw Milk

**DOI:** 10.3390/foods14030485

**Published:** 2025-02-03

**Authors:** Jintana Pheepakpraw, Chanita Sinchao, Sawannee Sutheeworapong, Pachara Sattayawat, Aussara Panya, Yingmanee Tragoolpua, Thararat Chitov

**Affiliations:** 1 Department of Biology, Faculty of Science, Chiang Mai University, Chiang Mai 50200, Thailand; jintana_ph@cmu.ac.th (J.P.); chanita_sin@cmu.ac.th (C.S.); pachara.sattayawat@cmu.ac.th (P.S.); aussara.pan@cmu.ac.th (A.P.); yingmanee.t@cmu.ac.th (Y.T.); 2 Doctor of Philosophy Program in Applied Microbiology (International Program), Faculty of Science, Chiang Mai University, Chiang Mai 50200, Thailand; 3 Systems Biology and Bioinformatics Laboratory, Pilot Plant Development and Training Institute, King Mongkut’s University of Technology Thonburi, Bangkok 10150, Thailand; sawannee.sut@kmutt.ac.th; 4 Environmental Science Research Center (ESRC), Faculty of Science, Chiang Mai University, Chiang Mai 50200, Thailand

**Keywords:** *Bacillus cereus* group, emetic toxin, cereulide, genome sequencing, dairy, food safety

## Abstract

The *Bacillus cereus* group frequently contaminates milk and dairy products. Some members of this group can produce the heat-stable pre-formed toxin cereulide, which causes emetic foodborne intoxication. This study characterised emetic *B. cereus* group isolates from raw cow’s milk in the biochemical, genetic, and toxigenic aspects. Of the 158 *B. cereus* group isolates derived from 99 raw milk samples, 7 (4.43%) harboured *cereulide synthetase A* (*cesA*), which encodes a cereulide synthetase associated with the emetic phenotype. Heat-treated culture filtrates from the *cesA*-positive isolates demonstrated cytotoxicity to HepG2 and Caco-2 cells, resulting in cell viabilities of 32.22–36.57% and 44.41–47.08%, respectively. The cytotoxicity levels were comparable to those of the reference emetic strain, F4810/72 (alternately termed AH187). Genome analysis of a representative isolate, CSB98, revealed the complete *ces* gene cluster with additional virulence factors such as non-haemolytic enterotoxin, haemolysins and phospholipases, suggesting that the isolate could be both emetic and diarrhoeagenic. CSB98 exhibited a closer relationship to the type strain of *B. paranthracis* than to that of *B. cereus sensu stricto* (ATCC 14579). The genomes of CSB98 and AH187 were indistinguishable through OrthoANI analysis, but 13 variants were identified via SNP calling. These results affirm genetic conservation among the emetic traits.

## 1. Introduction

*Bacillus cereus* and its closely related species, collectively referred to as the *B. cereus* group, pose substantial risks to the food industry due to their capacity to form endospores, which confer resistance to the high-heat treatments employed in food processing. Certain members of the *B. cereus* group are recognised as foodborne pathogens that can induce two forms of foodborne illnesses: diarrhoea and vomiting [1]. Diarrhoeal illness occurs from foodborne toxicoinfection involving various protein enterotoxins, including haemolysin BL (Hbl), non-haemolytic enterotoxins (Nhe), and cytotoxin K (CytK). Emetic illness occurs from foodborne intoxication and is attributed to the emetic toxin cereulide, which is pre-formed in food and causes the symptoms of nausea and vomiting to develop shortly (within 5–6 h) after the ingestion of food contaminated with the toxin. In severe cases, cereulide can cause liver failure [2]. Cereulide is a cyclic peptide with a molecular weight of 1.2 kDa [2,3]. It is synthesised by the cereulide synthetase multi-enzyme complex, which is encoded and regulated by the *cereulide synthetase* (*ces*) genes, through a non-ribosomal peptide synthesis (NRPS) pathway [4].

Cereulide exhibits resistance to elevated temperatures (150 °C for 30 min or 100 °C for up to 2 h) and a wide pH range (2–10) [1,5,6]. Consequently, conventional pasteurisation methods (e.g., 63 °C for 30 min or 72–75 °C for 15–30 s) and sterilisation (e.g., 135–150 °C for 1–10 s) employed in dairy processing [7] are unlikely to eradicate cereulide. As such, the presence of an emetic *B. cereus* group strain in a batch of raw milk may lead to cereulide contamination in the resulting processed products. Thus, it is crucial to maintain minimal levels of this organism by implementing appropriate sanitation measures in both the farm environment and the milking process [8]. This can be accomplished through regular cleaning and disinfection of the farm area and milking equipment, adherence to good hygiene practices during milking, and effective waste management [9]. The appropriate storage of raw and pasteurised milk products is also essential to inhibit cereulide production [10].

*B. cereus* group species are widely distributed in the dairy farm environment [11], making their contamination in raw milk inevitable [12,13]. Research indicates that *B. cereus* contamination levels in raw milk range from 3.8 to 100% across various geographical regions [14,15]. Emetic strains were infrequently found in milk, with a prevalence of less than 1.0–3.8%, and were also rare among dairy plant isolates [16]. However, the emetic *B. cereus* group species present in raw milk have not been sufficiently investigated, especially concerning their biological activity and genomic features. This information is essential for assessing the risk of this pathogen in relation to dairy products. This study aimed to determine the proportion of emetic isolates within *B. cereus* group isolates derived from raw milk, characterise them both genetically and biochemically, assess their cytotoxicity, and investigate the genomic features of a representative isolate.

## 2. Materials and Methods

### 2.1. Sources of B. cereus Group Isolates

The *B. cereus* group isolates used in this study were obtained from raw cow’s milk samples from individual farms, which were acquired at collective tanks situated at dairy cooperatives in different areas of Chiang Mai, Thailand. Each sample was collected in two sterile 50 mL conical tubes (approx. 40 mL in each tube), stored on ice during transportation to the laboratory, kept refrigerated at 4 °C, and analysed within 24–48 h. To isolate *B. cereus* group species, each raw milk sample from the two tubes was combined and diluted (10^−1^ to 10^−3^) in Butterfield’s phosphate-buffered dilution water. A 100 μL portion of each dilution was then surface-spread on polymyxin pyruvate egg yolk mannitol-bromothymol blue agar (PEMBA) or BACARA agar (BioMérieux, Marcyl’Étoile, France) [17,18]. Presumptive *B. cereus* group colonies from the PEMBA were characterised by flat, turquoise to peacock blue colonies measuring 2–5 mm, surrounded by an egg yolk precipitation zone [18]. Presumptive *B. cereus* group colonies from the BACARA agar were orange-pink colonies measuring 2–3 mm, surrounded by an egg yolk precipitation zone. The isolation was partially reported in a study by Pheepakpraw et al. (2023) [19]. A total of 158 colonies were obtained from 99 positive samples out of the 288 samples analysed. The isolates were cultured on tryptic soy agar (TSA) (Difco, Detroit, MI, USA) with 0.6% yeast extract. Samples were maintained at 4 °C or preserved as frozen stock cultures in 70% (*v*/*v*) glycerol for subsequent analysis by multiplex polymerase chain reaction (PCR).

### 2.2. PCR Analysis

Genomic DNA was extracted from pure cultures of 158 presumptive *B. cereus* group isolates employing a modified cetyltrimethylammonium bromide (CTAB) extraction method [20]. A multiplex PCR was conducted, targeting the *motB* and *cesA* genes, as well as an *its* region. The *motB* gene encodes the flagellar motor protein B specific to the *B. cereus* group [21]. The *cesA* gene encodes cereulide synthetase A, which is specific to emetic *B. cereus* [4]. The *its* fragment is located in the internal transcribed spacer region, which is universal among bacteria, and served as an internal control [22]. The PCR primers are listed in Table 1. Each 25 μL reaction mixture contained 2.5 μL of 10 × Taq Buffer with KCl, 0.2 μM of each primer, 0.2 mM of each dNTP, 3 mM of MgCl_2_, 1 unit of Taq DNA polymerase (Thermo Fisher Scientific, Waltham, MA, USA), and 1 µL of DNA template (0.5–5 μg/μL). PCR was performed using a thermal cycler (Mastercycler, Eppendorf, Hamburg, Germany). The amplification steps included an initial denaturation at 95 °C for 5 min, followed by 35 cycles of denaturation at 95 °C for 30 s, primer annealing at 59 °C for 45 s, extension at 72 °C for 1 min and a final extension at 72 °C for 5 min. The PCR products were analysed using 1.5% (*w*/*v*) agarose gel electrophoresis, stained with ViSafe Green Gel Stain solution (Vivantis, Shah Alam, Malaysia) in 1 × Tris-Acetate-EDTA (TAE) buffer and visualised on an UltraSlim blue LED transilluminator (Labgene Scientific, Châtel-Saint-Denis, Switzerland).

### 2.3. Biochemical Tests of cesA-Positive B. cereus Group Colonies

The *cesA*-positive *B. cereus* group isolates were subjected to biochemical testing following the procedures outlined in the US Food and Drug Administration (US FDA)’s Bacteriological Analytical Manual [18]. The tests comprised motility, egg yolk reaction, nitrate reduction, Voges–Proskauer (VP) reaction, haemolysis, anaerobic glucose utilisation, acid production from mannitol, rhizoid growth and the detection of protein toxin crystals. The isolates were also subjected to a carbohydrate fermentation test using API50 CHB (BioMérieux, Marcyl’Étoile, France). The results were submitted to APIWEB/API 50CHB V4.1 “https://apiweb.biomerieux.com (accessed on 13 November 2024)” for identification based on carbohydrate profiles.

### 2.4. 16S rRNA Gene Sequencing

The *cesA*-positive *B. cereus* group isolates were subjected to PCR amplification of the *16S rRNA* gene using primers 27F (5′-AGA GTT TGA TCC TGG CTC AG-3′) and 1492R (5′-GGT TAC CTT GTT ACG ACT T-3′) [23]. Each reaction mixture contained 2.5 µL of 10 × Taq buffer with KCl, 0.2 µM of each dNTP, 3 mM of MgCl_2_, 0.5 µM of each primer, 1 unit of Taq DNA polymerase (Thermo Fisher Scientific, Waltham, MA, USA) and 1 µL of DNA template (0.5–5 μg/μL). The PCR was performed with an initial denaturation step at 94 °C for 5 min, followed by 35 amplification cycles of denaturation at 94 °C for 30 s, annealing at 56 °C for 35 s, extension at 72 °C for 1 min, and a final extension at 72 °C for 10 min. The PCR products were analysed through 1.0% *(w/v)* agarose gel electrophoresis and visualised as previously described. Amplicons were purified using a Vivantis PCR Clean-Up Kit (Vivantis, Shah Alam, Malaysia) according to the manufacturer’s instructions and sequenced by U2Bio Sequencing Service Co., Ltd. (Seoul, Republic of Korea). The obtained sequences were analysed with BLAST “https://www.ncbi.nlm.nih.gov/BLAST/ (accessed on 4 May 2024)” to determine the closest relatives.

### 2.5. Cytotoxicity Test

#### 2.5.1. Preparation of Heat-Treated Culture Supernatants from *B. cereus* Group Isolates

To perform the cytotoxicity test, the *cesA*-positive *B. cereus* group isolates were cultured for 24 h at 30 °C in skim milk medium (SMM, comprising 2.5% skim milk powder in water; Difco, Detroit, MI, USA) [24]. The cultures were then heated at 121 °C for 15 min and subsequently centrifuged for 10 min at 7000 rpm at 4 °C to precipitate the cells. The supernatant was filtered through a 0.22 µm polyethersulfone (PES) syringe filter (Labfil, Hangzhou, China), and the resulting filtrate was utilised for the cytotoxicity test (2.5.2). *B. cereus* F4810/72, a reference emetic strain, as well as *B. cereus* DSM 4384, a diarrhoeal (non-emetic) strain, served as the culture controls. SMM (50%) served as a control for the culture medium.

#### 2.5.2. Cytotoxicity Assay

The hepatocellular carcinoma cell line HepG2 (ATCC HB-8065) and human colorectal adenocarcinoma cell line Caco-2 (ATCC HTB-37) were cultured under standard conditions in Dulbecco’s Modified Eagle Medium supplemented with 10% heat-inactivated foetal bovine serum and penicillin-streptomycin (DMEM-FBS-Pen/Strep) (the medium and supplements are from Gibco, Thermo Fisher Scientific, Waltham, MA, USA). Cells were plated at a density of 1 × 10^4^ cells/mL in 96-well plates and incubated at 37 °C in a 5% CO_2_ incubator for 24 h. The cell culture medium was discarded, and the cells were exposed to 100 µL of the heated culture supernatant from bacterial isolates/strains, diluted to 50% (*v*/*v*) with DMEM-FBS-Pen/Strep, for 24 h. Following the treatment, PrestoBlue reagent (Thermo Fisher Scientific, Waltham, MA, USA) was introduced, and the cells were incubated for 30 min at 37 °C in a 5% CO_2_ incubator [25]. Absorbance was measured at 570/600 nm using a BioTek Synergy HTX microplate reader (Agilent Technologies, Santa Clara, CA, USA). The percentages of cell viability in comparison to the non-treatment control (DMEM-FBS-Pen/Strep) were determined using the following equation [26]:

% Cell viability = [(OD_570_ − OD_600_) treated cells/(OD_570_ − OD_600_) non-treated cells] × 100

### 2.6. Genome Analysis of a cesA-Positive B. cereus Group Isolate

#### 2.6.1. Genome Sequencing, Assembly and Annotation

The genome of *Bacillus* sp. CSB98 (a *cesA*-positive *B. cereus* group isolate from raw milk) was sequenced using the Illumina NextSeq 550 System. Paired-end DNA libraries for Illumina sequencing were prepared according to the manufacturer’s recommendations using the Illumina DNA Library Prep Kit (Illumina Inc., San Diego, CA, USA). Sequencing was conducted by U2Bio Sequencing Service Co., Ltd. (Seoul, Republic of Korea). The quality of Illumina paired-end reads was evaluated using FastQC (version 0.12.1) [27]. The adapters and low-quality bases were trimmed using Trimmomatic software (version 0.39) [28]. Genome assembly was conducted using Unicycler (version 0.5.0) “https://github.com/rrwick/Unicycler (accessed on 30 October 2023)” [29], followed by genome annotation through the NCBI Prokaryotic Genome Annotation Pipeline (PGAP) [30] to analyse protein-coding sequences, RNA genes and pseudogenes. The genome assembly is available in the NCBI database (accession no.: JAXBCW000000000.1) under BioProject PRJNA1042944 (BioSample SAMN38327476).

#### 2.6.2. Analysis of the Major Functions of CSB98 in Comparison to Related Strains

The genome sequences of *Bacillus* sp. CSB98 were analysed in comparison to related strains available in the NCBI database. These strains included *B. cereus* ATCC 14579 (a type strain and diarrhoeagenic strain; accession no. GCA_006094295.1), *B. cereus* AH187 (F4810/72; an emetic strain isolated from vomit; accession no. GCA_000021225.1), and *B. paranthracis* Mn5 (an environmental strain originally isolated from Pacific Ocean sediments; accession no. GCA_001883995.1). The genome sequences were submitted to the Rapid Annotation using Subsystems Technology (RAST) server [31] for the annotation of key structural and metabolic subsystems. The RAST annotation results were visualised using the ggplot2 package in R (version 3.4.4) “https://ggplot2.tidyverse.org/ (accessed on 18 December 2024)” [32].

#### 2.6.3. Analysis of Virulence Factors, Antibiotic Resistance and Secondary Metabolites

Virulence factors related to enteropathogenicity were predicted from the assembled genome sequence of *Bacillus* sp. CSB98 using the Virulence Factor Database (VFDB) via the VFanalyzer pipeline [33] “https://www.mgc.ac.cn/VFs/ (accessed on 5 May 2024)”. Antibiotic resistance genes were analysed using the Comprehensive Antibiotic-Resistant Database (CARD) [34] “https://card.mcmaster.ca/ (accessed on 6 May 2024)”. Additionally, the biosynthetic gene clusters for secondary metabolites were analysed using the antiSMASH web server (version 7.0.1) [35] “https://antismash.secondarymetabolites.org/ (accessed on 19 July 2024)”.

#### 2.6.4. Analysis of Strain Relatedness

The genome sequences of *Bacillus* sp. CSB98 and 27 representative *B. cereus* group strains, obtained from the NCBI database, were analysed for their relatedness using the Type (Strain) Genome Server (TYGS) “https://tygs.dsmz.de (accessed on 20 December 2024)” [36]. In the analysis, AH187 and ATCC 14579 were selected as representatives of emetic and diarrhoeagenic strains, respectively. Pairwise genome comparisons were performed using the trimming algorithm and the distance formula *d*_5_ [37]. A genome BLAST distance phylogeny (GBDP) tree was constructed using FastME (version 2.1.6.1) http://www.atgc-montpellier.fr/fastme/ (performed via TYGS on 20 December 2024)”, incorporating SPR postprocessing [38]. Branch support values were determined using 100 pseudo-bootstrap replicates, and the tree was midpoint-rooted [39] and visualised using iTOL (version 7) “https://itol.embl.de/ (accessed on 16 January 2025) [40].

The Orthologous Average Nucleotide Identity (OrthoANI) values for strains closely related to *Bacillus* sp. CSB98 were calculated using the standalone Orthologous Average Nucleotide Identity Tool (OAT) software (version 0.93.1, Chunlab Inc., Seoul, Republic of Korea) [41]. Single-nucleotide polymorphisms (SNPs) were identified for CSB98 via Snippy (version 4.6.0) “https://github.com/tseemann/snippy (accessed on 21 January 2025)” [42], using AH187 as the reference genome.

## 3. Results and Discussion

### 3.1. Screening of B. cereus Group and Emetic B. cereus Group Species in Raw Milk

All 158 presumptive *B. cereus* group colonies recovered from raw milk samples were gram-positive, spore-forming, rod-shaped bacteria. All isolates were *motB*-positive, thereby confirming their identity as *B. cereus* group species. Seven of these isolates (4.43%) tested positive with the *cesA* primers, indicating that they had the potential to produce cereulide.

This proportion is comparable to those from previous studies conducted in Sweden, Japan and China, which report the proportions of emetic isolates in the total isolates from raw milk to be 1.5, 0.3 and 0.5%, respectively [16,43,44]. However, a survey in Ghana showed a much higher proportion (20.8%) [45]. In addition to previous research, the findings from our study suggest that emetic *B. cereus* group species, while infrequent, are distributed across various geographical regions, with raw milk identified as one of their food sources. Considering the heat resistance of their spores and the cereulide toxin [14], the potential for emetic foodborne intoxication linked to dairy products should not be overlooked.

### 3.2. Biochemical and Genetic Characterisation of cesA-Positive B. cereus Group Isolates

Seven *cesA*-positive *B. cereus* group isolates (CSB90, CSB91, CSB95, CSB96, CSB97, CSB98 and CSB100) underwent biochemical testing. All seven isolates exhibited positive results for catalase activity, motility, nitrate reduction, tyrosine decomposition, lysozyme resistance, egg yolk reaction, anaerobic glucose utilisation, VP reaction and haemolysis. Notably, all isolates failed to produce acid from mannitol. All isolates exhibited no formation of toxin crystals or rhizoid growth. Moreover, the biochemical characteristics align with those of *B. cereus*, as per the differentiation criteria outlined in the US FDA’s Biological Analytical Manual [18]. The API 50 CHB test indicated that the isolates exhibit carbohydrate fermentation profiles consistent with *B. cereus*, demonstrating an 86.0% identity (Table 2).

The isolates then underwent *16S rRNA* gene sequencing. The BLAST results indicate that the *16S rRNA* sequences of all *cesA*-positive milk isolates (NCBI accession no. PP981351–PP981357) were identical to those of several *B. cereus* group species in the NCBI database. The list comprises *B. cereus*, *B. thuringiensis*, *B. paramycoides*, *B. anthracis*, *B. tropicus*, *B. paranthracis*, *B. albus*, *B. toyonensis and B. nitratireducens*. The high genetic similarity among many species in the *B. cereus* group renders *16S rRNA* gene sequences inadequate for species-level identification, as acknowledged by previous studies [46,47,48]. The differentiation of *B. cereus* group species continues to depend on a polyphasic approach [49].

### 3.3. Cytotoxicity of cesA-Positive B. cereus Group Isolates to Caco-2 and HepG2 Cells

**Figure 1:**
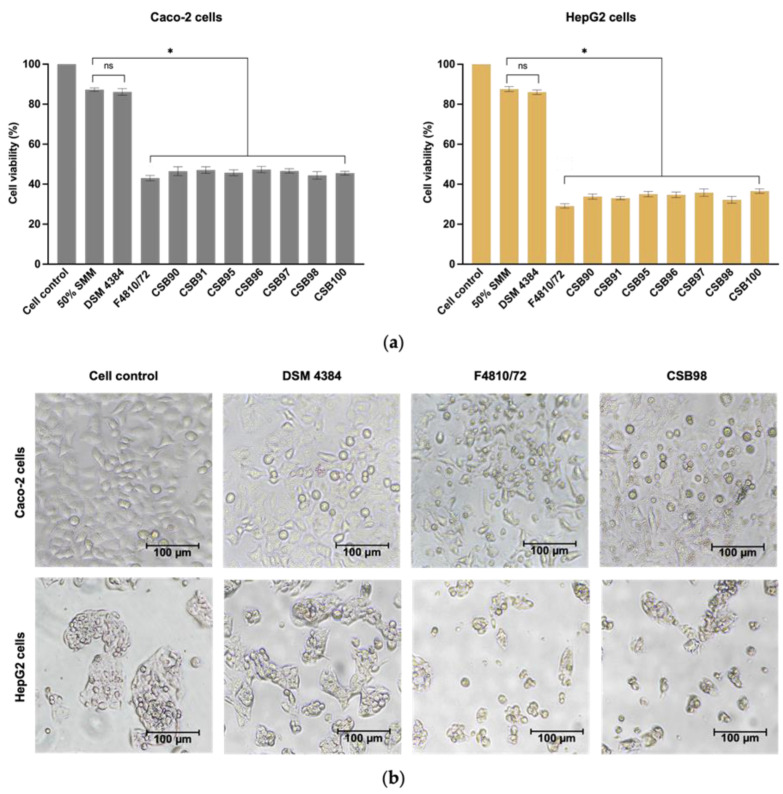
Viability (**a**) and changes in the morphology (**b**) of Caco-2 and HepG2 cells after treatment with heated culture filtrates of *cesA*-positive *B. cereus* group isolates (CSB isolates) compared to the culture medium control (50% SMM). DSM 4384 and F4810/72 (alternatively termed AH187) were used as reference diarrhoeagenic and emetic strains, respectively. The graphs were plotted using GraphPad Prism (version 10.1.1; GraphPad Software, San Diego, CA, USA). Error bars show ± SD from the triplicate experiments. The statistical differences (*p* < 0.05, one-way ANOVA) in cell viability are indicated above the bars (*). ns = not significantly different.

The cytotoxic effects on Caco-2 and HepG2 cells were evaluated for seven *cesA*-positive *B. cereus* group isolates, along with reference diarrhoeal and emetic strains. Caco-2 and HepG2 exhibited viabilities of 87.26 and 87.60%, respectively, in 50% SMM (Figure 1a). A level of 80% or higher is accepted as non-cytotoxic, in accordance with the ISO 10993-5 standard [50].

The heat-treated culture filtrates (diluted to 50%) from *cesA*-positive *B. cereus* group isolates cultured in SMM showed significant cytotoxicity (*p* < 0.05) to both cell types when compared to 50% SMM, with a more pronounced effect observed on HepG2 cells. The survival rates of Caco-2 and HepG2 following treatment with culture filtrates from the isolates were 44.41 to 47.08% and 32.22 to 36.57%, respectively. The cytotoxicity observed was similar to that of strain AH187 (F4810/72), which demonstrated survival rates of 43.07% ± 1.31 for Caco-2 and 29.10% ± 1.12 for HepG2. *B. cereus* DSM 4384, a diarrhoeal strain that does not produce cereulide (tested negative for *cesA*), exhibited no toxicity to either cell (Figure 1a).

Observations were made regarding the morphological changes in both cells treated with the heat-stable toxin derived from the isolates. Caco-2 cells exhibited elongation and detachment accompanied by notable vacuolation, while HepG2 cells transitioned from an aggregated morphology to a rounded shape (Figure 1b).

The detection of the *cesA* gene and the cytotoxicity of the milk isolates, which was comparable to that of the emetic strain F4810/72, suggests that the heat-stable toxin produced by the isolates is likely cereulide. Cereulide exhibits significant cytotoxic effects on HepG2 cells, as documented in prior studies [51,52,53,54]. Notably, it is recognised as both an emetic and a hepatic toxin [55,56].

### 3.4. Characterisation of the Bacillus sp. CSB98 Genome

The preceding sections demonstrated that a single raw milk sample produced *cesA*-positive *B. cereus* group isolates, which exhibited identical biochemical characteristics and *16S rRNA* gene sequences, as well as comparable cytotoxicity patterns. Consequently, isolate CSB98 was chosen for additional genome analysis. Raw sequence data of 889.8 Mbp were generated, and an assembled sequence of approximately 5.5 Mbp was retrieved. The genome information obtained from the assembly and annotation is summarised in Table 3.

Table 3 indicates that the genome size, GC content, and number of coding sequences (CDS) of *Bacillus* sp. CSB98 fall within the typical range observed for *B. cereus*. Additionally, the genome size of approximately 5.5 Mbp is comparable to that of *B. cereus* ATCC 14579, the type strain, which measures 5.4 Mbp [57,58]. The genome sizes of clinical *B. cereus* strains typically range from 4.6 to 5.8 Mbp [59]. The GC content of CSB98 is 35.5% and the CDS count is 5478, both of which fall within the established ranges for *B. cereus* strains (GC content of 35 to 35.5% and CDS of 4442 to 6724) [60,61].

### 3.5. Comparison of the Primary Functions of CSB98 with Related Strains

**Figure 2:**
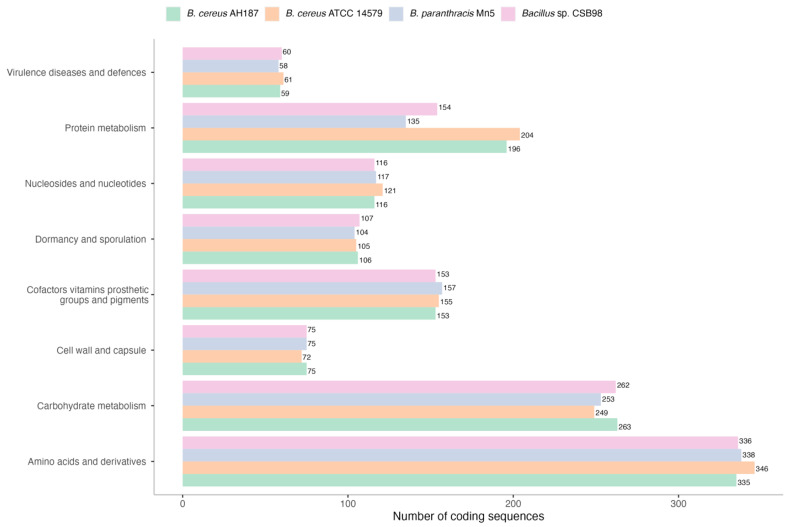
Overview of major functions identified in the genome sequence of *Bacillus* sp. CSB98, compared with related *B. cereus* group strains based on RAST annotation (http://rast.nmpdr.org) [31] and visualised using the ggplot2 package in R (version 3.4.4) “https://ggplot2.tidyverse.org/ (accessed on 18 December 2024)” [32]. Genome sequences of strains AH187 (an alternative name to F4810/72), ATCC 14579 and Mn5 were retrieved from the NCBI database.

The genes linked to different biological processes and metabolic pathways in the *Bacillus* sp. CSB98 genome were annotated and compared with those identified in related *B. cereus* group strains. The examined strains comprised a diarrhoeagenic strain (ATCC 14579), an emetic strain, (F4810/72; in this section, referred to as AH187, as it appears in the NCBI database) and a closely related strain (*B. paranthracis* Mn5). Similarities in the gene counts associated with various major metabolic categories were noted, particularly in the areas of amino acid, carbohydrate and micronutrient metabolism, which encompasses cofactors, vitamins, prosthetic groups and pigments. The structural functions, including nucleosides and nucleotides, dormancy and sporulation, and cell wall and capsule, exhibited significant similarity across the strains (see Figure 2). Additionally, the virulence and defence functions were also highly comparable among the strains. Although significant differences in protein metabolism were noted, these differences do not correlate with the classification of strains as emetic (CSB98 and AH187) or non-emetic (*ces*-negative) (ATCC 14579 and Mn5).

### 3.6. Analysis of the Virulence Factors

The virulence factors of *Bacillus* sp. CSB98 were analysed utilising the VFanalyzer pipeline from the VFDB [33]. The findings (Table 4) indicated that the CSB98 isolate contained all genes (*cesA*, *cesB*, *cesC*, *cesD*, *cesH*, *cesP* and *cesT*) typically found in the *ces* gene cluster. The entire cluster is considered crucial for cereulide virulence [4]. The deduced amino acid sequences were identical to those of the reference emetic strains (AH187 and NC7401).

Additionally, *Bacillus* sp. CSB98 harboured the *nhe* genes that encode non-haemolytic enterotoxin (Nhe), which is associated with diarrhoeal illness. The Nhe subunit sequences demonstrated complete identity with the reference emetic strains AH187 and NC7401. In contrast, their per cent identities were lower when compared to the subunit sequences of the reference Nhe-producing strain NVH1230-88, which exhibited identity values of 97.41, 99.75, and 95.82% for NheA, NheB and NheC, respectively. Given that Nhe is a significant diarrhoeal toxin, it suggests that the foodborne emetic *Bacillus* isolate CSB98 may also possess diarrhoeagenic properties. It is devoid of the *hbl* and *cytK* genes, which are responsible for encoding the enterotoxins haemolysin BL and cytotoxin K. The toxin profile of CSB98 closely resembles that of previously documented emetic strains, including BCRC17039 and others, such as F4810/72, F3351/87, F3752A/86 and NC7401 [21,62], indicating a degree of genetic conservation among these strains.

Further analysis of virulence-related factors indicated that *Bacillus* sp. CSB98 contained genes encoding pore-forming haemolysins, specifically *alo* and *hlyIII*, which encode anthrolysin O and haemolysin III, respectively. These substances increase virulence through the lysis of various cell types [63]. The isolate also possesses genes that encode phosphatidylcholine-preferring phospholipase C (*plcA*), phosphatidylinositol-specific phospholipase C (*pipLC*) and sphingomyelinase (*sph*), all of which serve roles in the degradation of host cell membranes. The virulence factors, including pore-forming haemolysins, phospholipases and sphingomyelinase, augment the isolate’s virulence, aligning with the traits of pathogenic *B. cereus* [64]. The immune inhibitor A metalloproteinase and a polysaccharide capsule enable the evasion of macrophage ingestion and confer resistance to host immune responses [65,66].

*Bacillus* sp. CSB98 also possesses iron acquisition genes, including those responsible for bacillibactin synthesis (*dhbA*, *dhbB*, *dhbC*, *dhbE* and *dhbF*), as well as genes encoding heme-acquisition leucine-rich repeat protein (*hal*) and iron-regulated leucine-rich surface protein (*ilsA*). These features indicate a competitive advantage in iron-limited environments and the ability to adapt to the iron-deficient conditions of the digestive tract [67]. Furthermore, the regulatory genes *plcR* and *papR*, which encode a transcriptional regulator and a cell–cell signalling peptide, respectively, coordinate the expression of extracellular virulence factors in the *B. cereus* group [68]. Notably, these genes may influence the expression of virulence factors and the pathogenicity of the strain [69].

In addition to virulence factors, antibiotic resistance was determined using CARD [34]. The antibiotic resistance factors discovered in *Bacillus* sp. CSB98 included genes encoding fosfomycin thiol transferase (*fosB*), class A *Bacillus cereus* beta-lactamase (*bcI*) and subclass B1 *Bacillus cereus* beta-lactamase (*bcII*). The *fosB* gene is responsible for resistance to fosfomycin—a phosphonic acid antibiotic that inhibits cell wall synthesis and is commonly used to treat uncomplicated urinary tract and gastrointestinal infections [70]. The *bcI* and *bcII* genes are associated with resistance to beta-lactam antibiotics due to beta-lactamase production [71]. *B. cereus* is typically resistant to beta-lactam antibiotics, and beta-lactamase production can confer resistance to penicillin, ampicillin and some third-generation cephalosporins [72].

### 3.7. Analysis of Secondary Metabolites

Secondary metabolites in *Bacillus* sp. CSB98 were identified using antiSMASH (version 7.0.1) [35]. In addition to cereulide and bacillibactin (see Table 4), other identified functional peptides include fengycin and thailanstatin A. Fengycin is a lipopeptide recognised for its efficacy against filamentous fungi [73]. This peptide has been identified in *Bacillus* species, including *B. cereus* Z4 [74] and *B. velezensis* FZB42 [75]. Thailanstatin A, a natural product initially isolated from the bacterial strain *Burkholderia thailandensis* MSMB43, is recognised as a spliceosome inhibitor with considerable potential in anticancer therapy [76]. It has been identified in *Bacillus* species, including *B. paranthracis* MHSD3 [77] and *B. subtilis* MBB3B9 [78].

### 3.8. Analysis of Strain Relatedness

**Figure 3:**
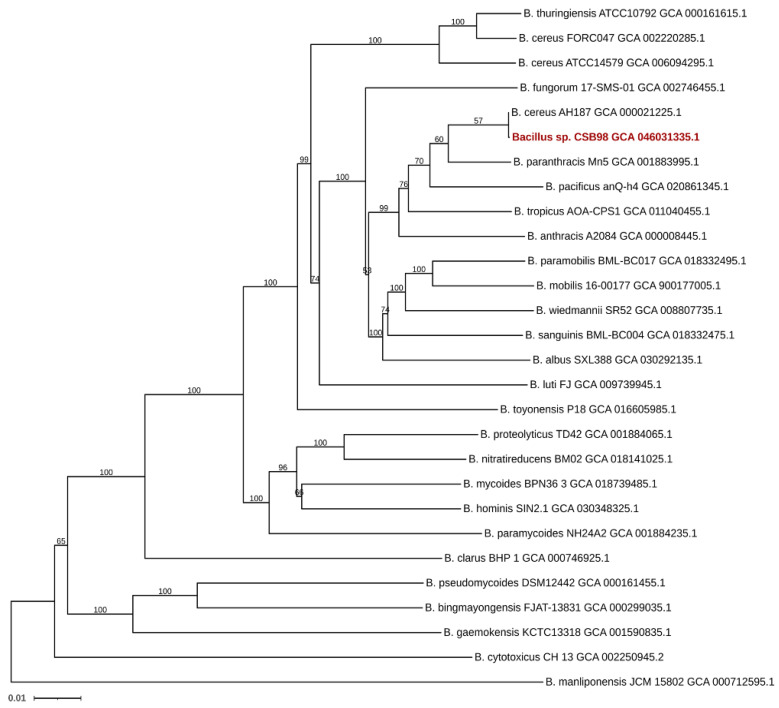
Phylogenomic tree of *Bacillus* sp. CSB98 (shown in red) and 27 representative strains of the *B. cereus* group species constructed using the Type Strain Genome Server (TYGS) (https://tygs.dsmz.de/ (accessed on 20 December 2024)” 36]. Tree inferred using FastME 2.1.6.1 “http://www.atgc-montpellier.fr/fastme/ (performed via TYGS on 20 December 2024)” [38] based on GBDP distances calculated from genome sequences obtained from the GenBank database. The branch lengths are scaled in terms of the GBDP distance formula *d*_5_ [37]. The numbers at the branches are GBDP pseudo-bootstrap support values > 60% from 100 replications, with an average branch support of 87.6%. The tree was rooted at the midpoint [39] and visualised using iTOL [40].

**Figure 4:**
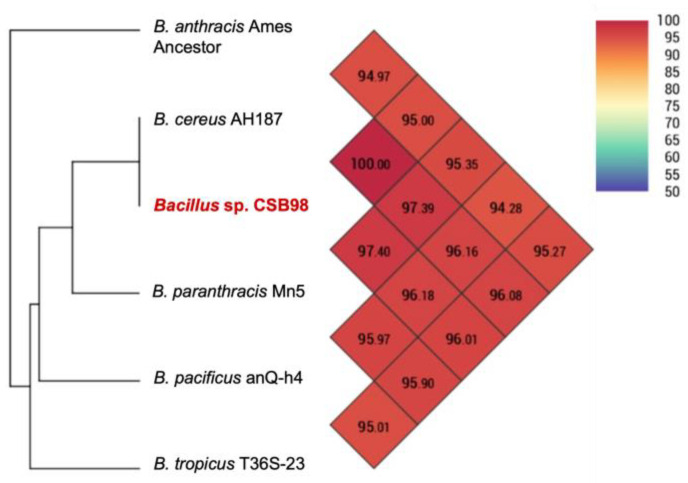
ANI values of *Bacillus* sp. CSB98 (shown in red) and closely related *B. cereus* group species, calculated using the Orthologous Average Nucleotide Identity Tool (OAT) software (version 0.93.1) [41].

The genomic relatedness of *Bacillus* sp. CSB98 to 27 representative strains in the *B. cereus* group was analysed, and a phylogenomic tree was constructed (Figure 3). The strains closely associated with CSB98 were subjected to additional analysis for ANI (Figure 4). The tree and ANI values indicate that *Bacillus* sp. CSB98 and the cereulide-producing *B. cereus* group strain AH187 (F4810/72) are indistinguishable at the resolution employed in the analysis. Additional analysis of the genomic variation between CSB98 and AH187 revealed 13 variants through alignment and variant calling, including one complex variant, four deletions, two insertions and six SNPs. These results indicate that the strains are likely not identical, yet they support the genetic conservation noted among different emetic *B. cereus* group strains [79].

CSB98 and AH187 demonstrated the highest genetic similarity to the type strain of *B. paranthracis* (Mn5), significantly surpassing that of the type strain of *B. cereus* (ATCC 14579) (Figure 3). OrthoANI analysis indicated a high (97.40%) similarity between CSB98 and *B. paranthracis* Mn5—a finding also noted by Caroll et al. (2019), who highlighted a close relationship to several emetic (*cesABCD*-positive) *B. cereus* group strains [79].

The genome sequence of CSB98 has been deposited in the NCBI database. Genomic analysis reveals a close relationship with *B. paranthracis*, whereas polyphasic characterisation supports its general characteristics being linked to *B. cereus*. Concerns have been raised about the identification of *B. cereus* group species based solely on genomic data [80]. This study does not aim to identify the species of the CSB98 isolate, especially given the variability in the ability to induce emetic illness both within and among species. As an isolate from milk—a known food reservoir for the *B. cereus* group—the significant relationship of CSB98 with other members of this group, as well as its virulence factor profile, suggest that emphasis should be placed on its emetic potential and gastrointestinal-associated virulence functions. Overall, knowledge regarding the virulence potential of an emetic *B. cereus* group species is useful for safety monitoring in the dairy production chain.

## 4. Conclusions

This study found that seven out of one hundred fifty-eight *B. cereus* group isolates from raw milk contained the *cesA* gene, suggesting their potential to produce cereulide. Their *16S rRNA* sequences indicated their affiliation with *B. cereus* group species, and they had all the biochemical characteristics of typical *B. cereus*. The heat-treated culture filtrates from *cesA*-positive isolates grown in skim milk medium demonstrated cytotoxic effects on HepG2 and Caco-2 cells, leading to cell viabilities of 32.22–36.57% and 44.41–47.08%, respectively. The cytotoxicity levels of these seven milk isolates were comparable to those of the emetic *B. cereus* strain AH187 (F4810/72). Genome sequencing of the *cesA*-positive isolate CSB98 identified several key virulence factors, including cereulide synthetase, non-haemolytic enterotoxin, haemolysins, phospholipases and sphingomyelinase, indicating that this strain can be both emetic and diarrhoeagenic. Furthermore, the functional peptides bacillibactin and fengycin were identified. Genomic variation between the CSB98 isolate and its closest strain (AH187) was found via SNP calling. CSB98 exhibited a closer relationship to the type strain of *B. paranthracis* than to the type strain of *B. cereus sensu stricto* (ATCC 14579).

## Figures and Tables

**Table 1:** PCR primers for detection of emetic *B. cereus* group.

Target Gene	Primer	Reference	PCR Product Size (bp)	Primer Sequence (5′ to 3′)
*cesA*	*cesA* F	[19]	996	CCG CCA GCT AGA TGA AAA AGA
	*cesA* R			ATC ACT TTC GGC GTG ATA CC
*motB*	*motB* F	[21]	575	ATC GCC TCG TTG GAT GAC GA
	*motB* R			CTG CAT ATC CTA CCG CAG CTA
*its*	*its* F	[22]	185	AAT TTG TAT GGG CCT ATA GCT CAG CT
	*Its* R			TTT AAA ATA GCT TTT TGG TGG AGC CT

**Table 2:** API 50 CHB profile of seven *cesA*-positive *B. cereus* group isolates.

Classification	Positive Reaction	Negative Reaction
Monosaccharides	D-ribose, D-glucose, D-fructose	D-arabinose, L-arabinose, D-xylose, L-xylose, D-lyxose, D-galactose, D-mannose, L-sorbose, L-rhamnose, D-tagatose, D-fucose, L-fucose
Disaccharides	D-cellobiose, D-maltose, D-saccharose (sucrose), D-trehalose, D-turanose	D-lactose, D-melibiose, gentiobiose
Trisaccharides	-	D-melezitose, D-raffinose
Polysaccharides	-	Inulin, amidon (starch), glycogen
Sugar alcohols	Glycerol	erythritol, D-adonitol, dulcitol, inositol, D-mannitol, D-sorbitol, xylitol, D-arabitol, L-arabitol
Amino sugars and sugar derivatives	N-acetyl-glucosamine, potassium gluconate	Potassium 2-keto-gluconate, potassium 5-keto-gluconate
Methyl glycosides (methylated sugars)	-	Methyl-β-D-xylopyranoside, methyl-α-D-mannopyranoside, methyl-α-D-glucopyranoside
Glycosides and complex sugars	Arbutin, esculin ferric citrate, salicin	Amygdalin

**Table 3:** Genomic profile of *Bacillus* sp. CSB98.

Genome Information	Value
Genome assembly based on Unicycler	
Number of contigs	157
N50 (bp)	277,966
Total length (bp)	5,472,779
Genome annotation based on PGAP	
GC content (%)	35.5
Protein coding sequence (CDS)	5478
The number of rRNA (5S, 16S, 23S)	3, 1, 1
The number of tRNA	73
ncRNA	5
Pseudogenes (total)	174

**Table 4:** Virulence-related and antibiotic resistance factors identified in the genome of *Bacillus* sp. CSB98.

Class No.	Classification *	Virulence Factor	Related Gene
1	Gastrointestinal-related toxins	Cereulide	*cesA*, *cesB*, *cesC*, *cesD*, *cesH*, *cesP*, *cesT*
		Non-haemolytic enterotoxin (Nhe)	*nheA*, *nheB*, *nheC*
2	Cytolysins	Anthrolysin O	*alo*
		Haemolysin III	*hlyIII*
3	Enzymes	Immune inhibitor A metalloproteinase	*inhA*
		Phosphatidylcholine-preferring phospholipase C (PC-PLC)	*plcA*
		Phosphatidylinositol-specific phospholipase C (PI-PLC)	*piplC*
		Sphingomyelinase (SMase)	*sph*
4	Immune evasion	Polysaccharide capsule	Undetermined
5	Iron acquisition	Bacillibactin	*dhbA*, *dhbB*, *ahbC*, *dhbE*, *dhbF*
		Heme-acquisition leucine-rich repeat protein (Hal)	*hal*
		Iron-regulated leucine-rich surface protein (IlsA)	*ilsA*
6	Regulation	PIcR-PapR quorum sensing	*papR*, *pIcR*
7	Antibiotic resistance	Class A *Bacillus cereus* Bc beta-lactamase (BcI)	*bcI*
		Subclass B1 *Bacillus cereus* Bc beta-lactamase (BcII)	*bclI*
		Fosfomycin thiol transferase (FosB)	*fosB*

* Classes 1–6 were analysed using the Virulence Factor Database (VFDB) [33], while class no. 7 was analysed using the Comprehensive Antibiotic Resistance Database (CARD) [34].

## Data Availability

Publicly available datasets were analysed in this study. These data can be found in the NCBI database under the accession number PRJNA1042944.
